# Relationship Between Infiltration of CD163^+^ TAMs, FoxP3^+^ Tregs, or CD66b^+^ TANs and Cell Differentiation in Colorectal Cancer Tissues

**DOI:** 10.5152/tjg.2023.22064

**Published:** 2023-07-01

**Authors:** Xiaobo Wang, Yongyu Bai, Zhuha Zhou, Bailiang Ye, Zhejing Chen, Xiaolei Chen, Wenyi Wu

**Affiliations:** 1 Department of Traumatology, The First Affiliated Hospital of Wenzhou Medical University, Wenzhou, Zhejiang Province, China; 2 Department of Gastrointestinal Surgery, The First Affiliated Hospital of Wenzhou Medical University, Wenzhou, Zhejiang Province, China

**Keywords:** Colorectal cancer, CD163, FoxP3, CD66b, cell infiltration, differentiation

## Abstract

**Background/Aims:**

There are many studies on immune cell infiltration in colorectal cancer, including FoxP3^+^-regulatory T cells, CD66b^+^ tumor-associated neutrophils, and CD163^+^ tumor-associated macrophages. These studies mainly focus on the relationship between cell infiltration and tumor progression, prognosis, and so on, while the relationship between tumor cell differentiation and cell infiltration is poorly understood. We aimed to explore the relationship between cell infiltration and tumor cell differentiation.

**Materials and Methods:**

The tissue microarray and immunohistochemistry were used to determine the infiltration of FoxP3^+^-regulatory T cells, CD66b^+^ tumor-associated neutrophils, and CD163^+^ tumor-associated macrophages in 673 colorectal cancer samples from the Second Affiliated Hospital, Wenzhou Medical University (2001-2009). Kruskal–Wallis test was used to assess the positive cell infiltration in colorectal cancer tissues with tumor cells of varying degrees of differentiation.

**Results:**

The number of CD163^+^ tumor-associated macrophages, FoxP3^+^-regulatory T cells, and CD66b^+^ tumor-associated neutrophils in colorectal cancer tissues was different, and the level of CD163^+^ tumor-associated macrophages was the highest while the level of FoxP3^+^-regulatory T cells was the least. There were significant differences in the cell infiltration of colorectal cancer tissue cells with different levels of differentiation (*P* < .05). The highest infiltration of CD163^+^ tumor-associated macrophages (154.07 ± 6.95) and FoxP3^+^-regulatory T cells (20.14 ± 2.07) were in the poorly differentiated colorectal cancer tissues, while the higher infiltration of CD66b^+^ tumor-associated neutrophils was in the moderately or well-differentiated colorectal cancer tissues (36.70 ± 1.10 and 36.09 ± 1.06, respectively).

**Conclusion:**

Infiltration of CD163^+^ tumor-associated macrophages, FoxP3^+^-regulatory T cells, and CD66b^+^ tumor-associated neutrophils in colorectal cancer tissues may be related to the differentiation of tumor cells.

**Main Points**

- The infiltration levels of CD163^+^ tumor-associated macrophages in colorectal cancer tissues were higher than those of FoxP3^+^-regulatory T cells and CD66b^+^ tumor-associated neutrophils.
- The infiltration of CD163^+^ tumor-associated macrophages and FoxP3^+^-regulatory T cells is negatively correlated with the degree of colorectal cancer cells differentiation.
- CD66b^+^ tumor-associated neutrophils tend to infiltrate in moderately or well-differentiated colorectal cancer tissues.

## INTRODUCTION

The occurrence of malignant tumors is a multi-step complex process. Abnormal cell differentiation is one of the biological characteristics of cancer.^1^ During the process of benign lesions transforming to malignant tumors, the cells progressively acquiring the undifferentiated state by reversal of the differentiation signals is one of the critical changes.^2^ The abnormal differentiation of tumor cells is a complex process involving multiple links, multiple steps, and multiple targets.^3^ Current research on the mechanism of tumor cell differentiation is mostly focused on the regulation of oncogene and tumor suppressor gene expression, cell cycle regulation, cell signal transduction pathway regulation, and related enzyme activities.^4-6^

In the tumor microenvironment, the interaction between malignant tumor cells and immune cells exerts important function in invasion, metastasis, and recurrence of tumor.^7^ Immune cells interact with and cooperate with tumor cells in the tumor microenvironment, this determines the progression or regression of tumors.^8^ Compared with traditional indicators of prognosis evaluation, the prognostic predictive model established from the numbers of infiltrating regulatory T cells (Tregs), tumor-associated macrophages (TAMs), or tumor-associated neutrophils (TANs) facilitate the prognostic evaluation of colorectal cancer (CRC) patients.^9^ FoxP3^+^ Tregs belong to a class of T cell subtypes that express CD4, CD25, and FoxP3 in T lymphocytes. The abnormal development and dysfunction of Tregs cells are associated with a variety of major immune-related diseases, including transplant rejections, infectious diseases, allergic diseases, autoimmune diseases, and tumor immune tolerance.^10,11^ CD66b is the specific molecular marker on the surface of TANs cell membrane,^12^ the number of CD66b^+^ TANs infiltration in a variety of tumors is closely related to the prognosis of patients.^13^ Tumor-associated macrophages are formed by monocytes in the peripheral blood that invade tumor tissues, and play an important role in tumor growth, invasion, and metastasis.^14,15^ A previous study revealed that, in the tumor microenvironment of CRC, a mutually negative correlation was observed between CD66b^+^ TANs and CD163^+^ TAMs, and between CD66b^+^ TANs and FoxP3^+^ Tregs.^16^ So, in the tumor microenvironment, the infiltration of CD66b^+^ TANs, CD163^+^ TAMs, and FoxP3^+^ Tregs plays important roles.

Some studies have explored the relationship between tumor cell differentiation and the infiltration of FoxP3^+^ Tregs, CD66b^+^ TANs, or CD163^+^ TAMs in the tumor microenvironment. For example, a previous study found that the infiltration levels of intraepithelial FoxP3^+^ cells in poor or undifferentiated carcinomas were higher.^17^ In gastric cancer, high infiltration of CD163^+^ TAMs and CD66b^+^ TANs are significantly related to poor differentiation and well differentiation, respectively.^18^ However, we did not found studies that systematically and simultaneously explored the effects of the degree of tumor cell differentiation on infiltration of FoxP3^+^ Tregs, CD66b^+^ TANs, or CD163^+^ TAMs in the tumor microenvironment of CRC.

In this study, we collected CRC samples with different levels of differentiation, detected the infiltration of FoxP3^+^ Tregs, CD66b^+^ TANs, and CD163^+^ TAMs, and analyzed the relationship between cell infiltration and tumor cell differentiation.

## MATERIALS AND METHODS

### Patients

The medical-ethical approval was acquired from the ethics committee of the Second Affiliated Hospital, Wenzhou Medical University and informed consent was also gained from each patient. A total of 673 CRC samples from the Second Affiliated Hospital, Wenzhou Medical University (2001-2009) were retrospectively collected. The inclusion criteria for cases to be studied included the following: (1) primary CRC without other coexisting malignancies, (2) patients were confirmed as CRC by 2 pathologists combing biopsy with postoperative pathological diagnosis, and (3) availability of tissue blocks for investigation. The exclusion criteria cases to be studied were patients with previous history of radiotherapy, chemotherapy, and immunity therapy. We also collected the clinicopathological features of patients. Tumor node metastasis (TNM) stages were classified according to the American Joint Committee on Cancer guideline (7th Edition).

### Tissue Microarray and Immunohistochemistry

After fixed with formalin, all the samples were processed into paraffin-embedded tissues. All paraffin-embedded tissues were sliced to performed hematoxylin and eosin stain, then the typical lesion was selected under the light microscope, and the location of typical lesion was marked in the corresponding paraffin tissue blocks. The diameter of 1 mm paraffin tissue block was took out of paraffin tissue blocks by a hole puncher and inserted into the perforated receptor paraffin block for constructing tissue microarray. After being sliced into 4 μm sections, the tissue microarray was performed for immunohistochemistry as described previously.^19^ After dewaxing, rehydrating, and blocking endogenous peroxidase, the tissue array was used for antigen retrieval with sodium citrate antigen retrieval buffer (pH = 6.0, 10 mM). Tissue arrays were incubated with primary antibody for CD66b (ab197678, Abcam, at a dilution of 1:100), FoxP3 (MAB8214, BD Biosciences, at a dilution of 1:200), and CD163 (ab182422, Abcam, at a dilution of 1:500) at 4°C overnight. Then the tissues were incubated with horseradish peroxidase (HRP)-conjugated goat anti-rabbit secondary antibody (ab97080, Abcam; at a dilution of 1:2000) or HRP-conjugated goat anti-mouse secondary antibody (ab97040, Abcam; at a dilution of 1:500) at room temperature. Then the slices were stained with diaminobenzidine and counterstained with hematoxylin. Positive staining was calculated using Image Pro plus 6.0 (Media Cybernetics Inc, Bethesda, MD) under high power filed (HPF, 200) in 3 different fields of view in per slice.

### Clinical Parameters of Outcomes and Statistical Basis

The age distribution was transformed by categorical variable with 60 years as the cutoff value. The carbohydrate antigen 19-9 (CA19-9) and carcinoembryonic antigen (CEA) values were also divided into 2 groups with the normal values of 5.0 ng/mL and 37 U/mL as the cutoff values.^20^ These data were shown as n (%), and comparison of clinical characteristics between different groups were performed by chi-square test. Non-normally distributed variable data were expressed as mean ±  SE. Kruskal–Wallis rank sum test and a post hoc Dunn’s test were used to evaluate the positive cell infiltration in CRC tissues with tumor cells of varying degrees of differentiation. All statistical analyses were performed using statistical package SPSS (version 22.0 for Windows) (IBM Corp.; Armonk, NY, USA) and *P* < .05 was considered statistically significant.

## RESULTS

Among the 673 samples, the number of samples with moderately differentiated cells was the largest (640), while the number of samples with highly differentiated cells was the least, with only 5. There were no significant differences in age and gender distribution, tumor location, serum levels of CA19-9 and CEA between groups with different degrees of differentiation. There were significant differences in TNM staging between different groups. Patients with poorly differentiated samples were mostly in TNM stage III (67.9%), while patients with moderately differentiated samples were mostly in TNM stage II (43.0%) (Table 1).

**Figure 1.**
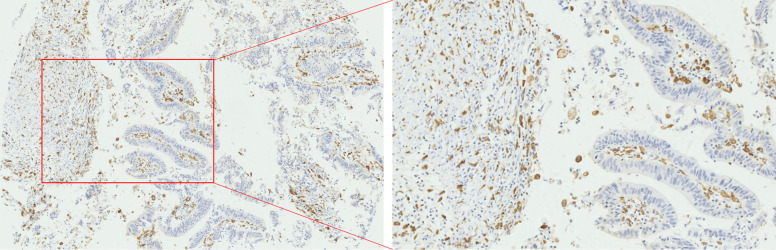
Immunohistochemistry for CD163^+^ TAMs infiltration in CRC tissue.

**Figure 2.**
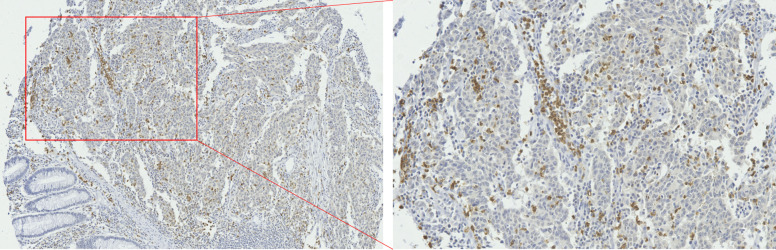
Immunohistochemistry for CD66b^+^ TANs infiltration in CRC tissue.

**Figure 3.**
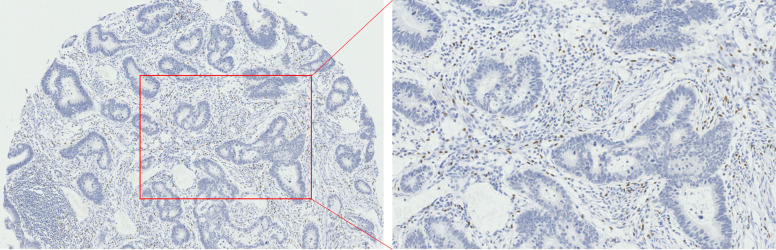
Immunohistochemistry for FoxP3^+^ Treg infiltration in CRC tissue.

The results of immunohistochemistry showed that among the 3 immune cells infiltrated in CRC tissues, the most infiltrated cell was CD163^+^ TAMs (Figure 1), followed by CD66b^+^ TANs (Figure 2), and the least infiltrated cell was FoxP3^+^ Tregs (Figure 3). With the changes in the degree of differentiation of tumor cells in CRC tissues, the number of FoxP3^+^ Tregs, CD163^+^ TAMs, and CD66b^+^ TANs cells changed significantly (*P* < .05). The number of FoxP3-positive cells in poorly differentiated CRC was largest (20.14 ± 2.07), which was significantly higher than that of moderately differentiated and well-differentiated CRC tissues (14.86 ± 0.28 and 15 ± 3.73) (*P* < .05). Similarly, the poorly differentiated CRC tissues recruited the most CD163-positive cells (154.077 ± 6.95), and the well-differentiated CRC tissue recruited the least CD163-positive cells (84.60 ± 13.20). The distribution of CD66b-positive cells was contrary to the distribution of FoxP3^+^ Tregs and CD163^+^ TAMs. CD66b^+^ TANs mainly infiltrated in CRC tissues with a higher degree of differentiation (moderately and well differentiated) (36.70 ± 1.10 and 36.09 ± 1.06), while lowly infiltrated in poorly differentiated CRC tissues (22.54 ± 4.24) (Table 2).

## DISCUSSION

Immune cell and inflammatory cell infiltration are mostly used in the prognosis prediction of various tumors, but the relationship between tumor cell differentiation and immune cell infiltration is not very clear.^18,21,22^ In this study, the infiltration of FoxP3^+^ Tregs, CD163^+^ TAMs, and CD66b^+^ TANs in CRC tissues was studied using tissue microarray and immunohistochemistry. The infiltrated FoxP3^+^ Tregs were the least cell compared with CD163^+^ TAMs and CD66b^+^ TANs in CRC tissues. In normal, the number of Foxp3^+^ Tregs is far less than macrophages and neutrophils. But the tumor microenvironment is complex, many factors lead to the infiltration of Tregs, TAMs, and TANs in tumor microenvironment. For example, TAMs express CCL18 to recruit naive CD4^+^ T cells developing into Tregs in tumor microenvironment.^23^ Therefore, the factors that result in different levels of infiltrating FoxP3^+^, Tregs CD163^+^ TAMs, and CD66b^+^ TANs are not only the number of these cells in normal condition. In future, we will explore the factors that infiltrating FoxP3^+^, Tregs CD163^+^ TAMs, and CD66b^+^ TANs.

Poorly differentiated CRC cells are discohesive and can secrete mucus to form a large amount of mucus/colloid. The mucus inside the tumor cell can push the nucleus to the cell membrane. And moderate differentiated CRC cells describe irregular tubular structures, harboring stratification, multiple lumens, reduced stroma. Well-differentiated CRC cells describe characteristics similar to normal cells.^24^ For the body’s immune system, poorly differentiated CRC cells are more heterogeneous than moderately or well-differentiated CRC cells, and are more prone to inflammatory and immune responses. An increased density of intraepithelial infiltration FoxP3^+^ cells was observed in poor or undifferentiated carcinomas.^17^ In our study, the infiltration levels of FoxP3^+^ Tregs in poorly differentiated CRC tissues were higher than those in moderately and well-differentiated CRC tissues. This may be due to FoxP3^+^ Tregs are known to have the function of suppressing immune response and maintaining immune tolerance.^25,26^ Besides, compared with moderately and well-differentiated CRC tissues, the numbers of infiltrating CD163^+^ TAMs in poorly differentiated CRC tissues were higher. In CRC, CD163 protein expression is dramatically positively related with tumor differentiation.^27^ However, a previous study indicated that there was no significant difference in CRC cell differentiation between the high-level CD163^+^ TAM infiltration and low-level CD163^+^ TAM infiltration groups.^28^ In addition, high infiltration of CD163^+^ TAMs is related to poor differentiation in gastric cancer.^18^ In patients with poorly differentiated HCC, the number of CD163^+^ cells is promoted.^29^ The infiltration of CD163^+^ TAMs in tumor tissues is related to anti-inflammatory.^30^ High levels of CD66b^+^ TANs in gastric cancer and CRC are related to a good prognosis,^31,32^ indicating that CD66b^+^ cells may play an anti-tumor effect in these tumor tissues.^33^ In our study, the lower levels of CD66b^+^ TANs in poorly differentiated CRC tissues were found. The density of infiltrating TANs is negatively related to the tumor differentiation in the lung adenocarcinoma.^34^ High infiltration of CD66b^+^ TANs is obviously associated with well differentiations in gastric cancer.^18^ Whether the low-level TANs in poorly differentiated tissues in this study is related to the anti-immunity and immune tolerance caused by CD163^+^ TAMs and FoxP3^+^ Tregs still needs further research to determine.

Many researches have reported the relationship between survival and the cell infiltration of CRC. The overall survival of CRC patients with CD163^+^ TAM infiltration in a low-level was longer.^28^ A high FoxP3^+^ Treg cell density in CRC tissues was correlated with better overall survival.^35^ In addition, previous studies reported that highly infiltrated FoxP3^+^ Treg cells were related to improved relapse-free survival.^36,37^ Colorectal cancer patients with fewer CD66b^+^ TANs had favorable relapse-free survival and overall survival.^38^ However, a previous study showed that high infiltration of these cells was associated with the short survival of CRC patients.^9^ So, we need to further explore the relationship between survival and the cell infiltration of CRC in the future.

We collected samples in the Second Affiliated Hospital, Wenzhou Medical University (2001-2009), but the samples with the highly differentiated cells were only 5. This is the limit in our study. However, the collection of clinical samples is a difficult. In addition, the acquisition of poorly differentiated samples may be more difficult. In future, we will collect more CRC samples to confirm the infiltrations of CD66b^+^ TANs, Foxp3^+^ Tregs, and CD163^+^ TAMs being associated with tumor differentiation.

## CONCLUSION

In this study, we indicate that the infiltration level of CD163^+^ TAMs in CRC tissues were highest compared with that of FoxP3^+^ Tregs and CD66b^+^ TANs. The infiltration levels of CD163^+^ TAMs may decrease with the increase of degree of CRC cells differentiation, which is similar to the infiltration trend of FoxP3^+^ Tregs in CRC tissues. Compared with poorly differentiated CRC tissues, CD66b^+^ TANs may be easier to infiltrate in moderately or well-differentiated CRC tissues.

## Figures and Tables

**Table 1.** Demographic and Baseline Characteristics in CRC Patients with Tumor Cells of Varying Degrees of Differentiation

Characteristics^a^	Differentiation			*P* ^b^
	Poor (%)	Moderate (%)	Well (%)	
*Age (years)*				.339
≤60	15 (53.600)	328 (51.300)	1 (20.000)	
>60	13 (46.400)	312 (48.800)	4 (80.000)	
*Gender*				.299
Male	13 (46.400)	391 (61.100)	3 (60.000)	
Female	15 (53.600)	249 (38.900)	2 (40.000)	
*Location*				.342
Rectum	14 (50.000)	407 (63.600)	3 (60.000)	
Colon	14 (50.000)	233 (36.400)	2 (40.000)	
*TNM stage*				.005
I	2 (7.100)	134 (20.900)	2 (40.000)	
II	7 (25.000)	275 (43.000)	3 (60.000)	
III	19 (67.900)	231 (36.100)	0 (0.000)	
*CEA*				.396
<5	16 (57.100)	412 (64.400)	2 (40.000)	
≥5	12 (42.900)	228 (35.600)	3 (60.000)	
*CA19-9*				.305
<37	22 (78.600)	556 (86.900)	5 (100.000)	
≥37	6 (21.400)	84 (13.100)	0 (0.000)	

CA, carbohydrate antigen; CEA, carcinoembryonic antigen; CRC, colorectal cancer; TNM, tumor node metastasis.

^a^n (%).

^b^Chi-square test.

**Table 2.** Immune Cells Infiltrated in the Microenvironment of Tumor Cells with Different Degrees of Differentiation

Indicator	Differentiation			*P* ^a^
	Poorly	Moderately	Well	
FoxP3^+^ Tregs	20.14 ± 2.07	14.86 ± 0.28	15 ± 3.73	0.013
CD163^+^ TAMs	154.07 ± 6.95	119.76 ± 1.96	84.60 ± 13.20	.000
CD66b^+^ TANs	22.54 ± 4.24	36.70 ± 1.10	36.09 ± 1.06	.001

^a^Kruskal–Wallis test.

TAM, tumor-associated macrophage; TAN, tumor-associated neutrophil.
